# Objections to the Inhalation of Solid Medicinal Substances, Considered

**Published:** 1849-07

**Authors:** M. M. Rogers


ART. V.—*Objections to the Inhalation of Solid Medicinal Substances, Considered.* By M. M. ROGERS, M. D.*

There are some objections to the mode of practice advocated in my former communication, which are entitled to consideration. The more important of these I will briefly notice.

*First Objection.*—The inhalation of solid substances, and particularly those which are insoluble, and which, therefore, would not be absorbed to any extent, would cause severe irritation and cough. This is true in some

* This was designed as a continuation of the article in the last No. of this Journal, but being received too late to be inserted in connection with that article, we now give it place as a distinct communication.

instances, as experience has proven,—but it by no means follows that this is the general consequence. Doct. Green, in his work on bronchitis, says, " mere opinions in medicine, however extensive the experience on other subjects, of those by whom they are entertained, cannot be received at the present day in opposition to facts." Prof. Lee says, in relation to cauterizing the larynx and trachea,—" here, as in many other cases, experience upsets all *a priori* reasoning,—the mistake arises from confounding the organic sensibility of the parts, that sympathetically causes cough, with the animal sensibility of this organ, which is extremely obtuse." We have evidence in the expectoration of men who work in a dense cloud of dust, as colliers and threshers, that insoluble matters may be inhaled to a large amount, and again expectorated without serious consequences.

*Second Objection.*—The function of the lungs is peculiar, and indispensable to life: and any cause which should chemically, mechanically, or physiologically interfere with aeration, respiration, nutrition, secretion, motion, organic or animal sensibility, must be injurious, or fatal. But this is true, to a greater or less extent, of every organ or system of organs, in the body. The crypts or follicles, and bronchial glands, differ in anatomy, though identical in functions, from the epiglottis to the extreme air cells of the lungs : but the mucous surface is, both in structure and function, all the way the same : it is nearly or entirely identical, also, with mucous membrane in all other parts.

*Third Objection.*—We do not know the quantity of any medicine which might be safely inhaled,—and our first trials might prove fatal to the patient. This is true, and could have been said once of the administration of every powerful medicine in use.

*Fourth Objection.*—Medicines inhaled act upon all parts of the lungs alike ; so that when disease is only local or partial, sound parts are unnecessarily attacked, and perhaps injured : but this objection holds true of all vaporized medicines ; and to the administration of medicines in the treat ment of nearly all diseases.

*Fifth Objection.*—Some medicines may produce dyspnœa, spasmodic asthma, or paralysis of the muscular fibres of the lungs,—and this last condition may be followed by asphyxia and death. To this objection it may be said, that extreme caution is necessary in making experiments. But, unless it is true that an infinitessimal dose might produce internal and even fatal results, we may commence safely with a dose almost as small, and increase at each succeeding one, up to the quantity which will show, by its effects upon the patient, how much may be safely given ; and this is the only way in which poisonous doses of any medicine can be known.

*Sixth Objection.*—That several of the irritating substances proposed to be inhaled, would be likely to produce inflammation. But we are all in the habit of prescribing these, and others much more powerful, both internally and externally, to mucous surfaces: in fact, all internal remedies must come in contact with mucous tissue. Besides this, the local application of some stimulant or irritant, quickens the circulation, and increases absorbent action. By this means, also, a temporary cough is produced, and strength given to the lungs, which results in the expectoration of mucus or other morbid deposites, which, from the feeble condition of the lungs, would otherwise remain, and blocking up the air passages, cause congestion, dyspnœa, &c. By this mode of stimulating the capillaries, the venous blood is hurried with increased velocity through the lungs, congestion relieved, and the blood better arterialized.

*Seventh Objection.*—Some medicines may act *chemically* in such manner as to interfere with aeration, or some other function. This is possibly true,—but experiment alone must determine. But it is almost certain that some medicines inhaled, may change chemically the alkaline, acrid, and other morbid characters of the pulmonary secretion, and, thereby removing a cause of local irritation, be followed by a cure. To conclude,—the superficial area of the mucus surface of the lungs is so great, and its sensibility so acute, that this means must prove highly beneficial in the medication of lung diseases. And I may be pardoned if I go still farther, and say, what may appear too dogmatical for one in my present position without facts, that I confidently believe inhalation will, when properly understood, be a remedy in lung diseases, excepting perhaps blood-letting, more powerful and controlling than all others combined.

